# How Much Is the Whole Really More than the Sum of Its Parts? 1 ⊞ 1 = 2.5: Superlinear Productivity in Collective Group Actions

**DOI:** 10.1371/journal.pone.0103023

**Published:** 2014-08-01

**Authors:** Didier Sornette, Thomas Maillart, Giacomo Ghezzi

**Affiliations:** 1 Department of Management, Technology and Economics, ETH Zurich, Zurich, Switzerland; 2 School of Information, UC Berkeley, Berkeley, California, United States of America; 3 Department of Informatics, University of Zurich, Zurich, Switzerland; University of Maribor, Slovenia

## Abstract

In a variety of open source software projects, we document a superlinear growth of production intensity (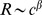) as a function of the number of active developers 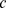, with a median value of the exponent 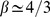, with large dispersions of 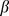 from slightly less than 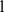 up to 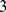. For a typical project in this class, doubling of the group size multiplies typically the output by a factor 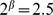, explaining the title. This superlinear law is found to hold for group sizes ranging from 5 to a few hundred developers. We propose two classes of mechanisms, *interaction-based* and *large deviation*, along with a cascade model of productive activity, which unifies them. In this common framework, superlinear productivity requires that the involved social groups function at or close to criticality, or in a “superradiance” mode, in the sense of the appearance of a cooperative process and order involving a collective mode of developers defined by the build up of correlation between the contributions of developers. In addition, we report the first empirical test of the renormalization of the exponent of the distribution of the sizes of first generation events into the renormalized exponent of the distribution of clusters resulting from the cascade of triggering over all generation in a critical branching process in the non-meanfield regime. Finally, we document a size effect in the strength and variability of the superlinear effect, with smaller groups exhibiting widely distributed superlinear exponents, some of them characterizing highly productive teams. In contrast, large groups tend to have a smaller superlinearity and less variability.

## Introduction

Since at least Aristotle, the adage in the title has permeated human thinking, with prominent influence in psychology (Gestalt theory [1]), biology (brain functions [2], ecological networks [3]), physics (spontaneous symmetry breaking [4] and the “more is different” concept [5]), economics [6], [7] among a wealth of other examples. Prominent among other developments are the fields of complexity science, synergetics and complex adaptive system theory, which strive to understand natural and social systems in terms of a systemic or holistic approach, where the above adage is translated into the scientific concept of *emergence* that results from repetitive interactions between simple constituting elements in extended out-of-equilibrium adaptive systems. Dealing with groups such as firms and production units, management science also strives to understand when and how a group can be more than the sum of individuals, and to design ways to improve team performance [8]–[11], through the mechanism of complementarity in organization [12], [13] and innovations [14]. Because most activities in our modern environment require coordination and collaborative actions within groups of widely varying sizes, it is the fundamental aspiration of any manager, be it in the public or private sector, to find the gears that could enhance productivity.

Notwithstanding their importance in human culture and civilization since ancient times, we still have a limited understanding of the mechanisms at the origin of group productivity. Moreover, we do not really understand the conditions under which the whole is more than the sum of its parts, and how to quantify its productivity with respect to its different constituents. The bottlenecks hindering progress include the difficulties for quantifying productivity as well as the obstacles of controlled experiments that allow for clean conclusions. Indeed, most human groups and systems are entangled in their functioning and objectives, and are rarely amenable to systematic and continuous observations suitable for rigorous scientific analyses.

To address these problems, we use a source of data in which group cooperation is ubiquitous and can be quantified in great details, namely the dynamics of production intensity during the development of open source software (OSS) projects. Because OSS development is essentially collective, iterative, and cumulative, and the overhead costs for interactions is small thanks to the cheap electronic support mediating exchanges between developers, the study of potential increases of productivity by interaction and cooperation between several contributing developers is particularly well suited.

The next section presents the main empirical evidence of the superlinear production intensity law found for open source software projects. We then present two classes of mechanisms at the origin of superlinear production intensity, which are unified in the cascade model of productive activity. Empirical data tests are found to support the model. We then compare and attempt to reconcile present findings for OSS and the superlinear law previously reported for cities. A discussion section develops the broader implications of our results, and the conclusion section summarises our main results.

## Quantification of productivity in open source software projects

**Figure 1:**
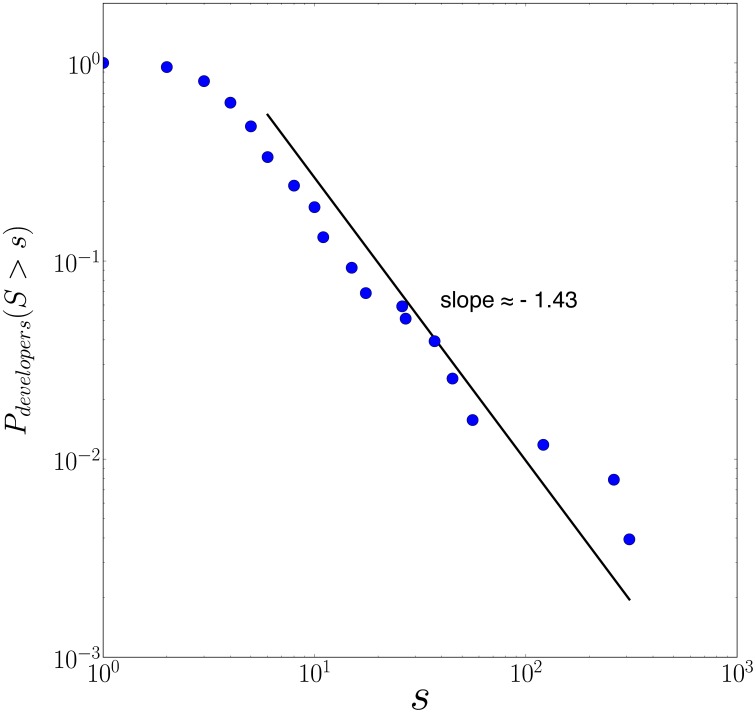
Distribution of project sizes in our sample quantified by their total number of developers. The distribution follows approximately a power law with exponent 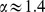, with an apparent deviation in the tail possibly resulting from an over-sampling bias of large projects. The bend down for small projects is likely the result of an under-sampling bias.

We have analyzed the production for 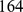 open source software projects of size ranging from 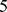 to 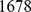 contributors. Figure 1 shows the complementary cumulative distribution of project sizes in our sample quantified by the number of developers involved in each project [all source data (Archive S1) and relevant statistics (Table S1), detailed per project, are available in Supporting Information]. The distribution is an approximate power law 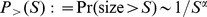 with exponent 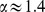, which reflects a large heterogeneity of project sizes with few projects attracting many developers and a multitude of projects with just a few developers. The simplest generic mechanism for such power law distribution of human group sizes is proportional growth coupled with birth and death [15], [16] as verified empirically in OSS package reuse [17], in group [18] and in product [19] dynamics.

**Figure 2:**
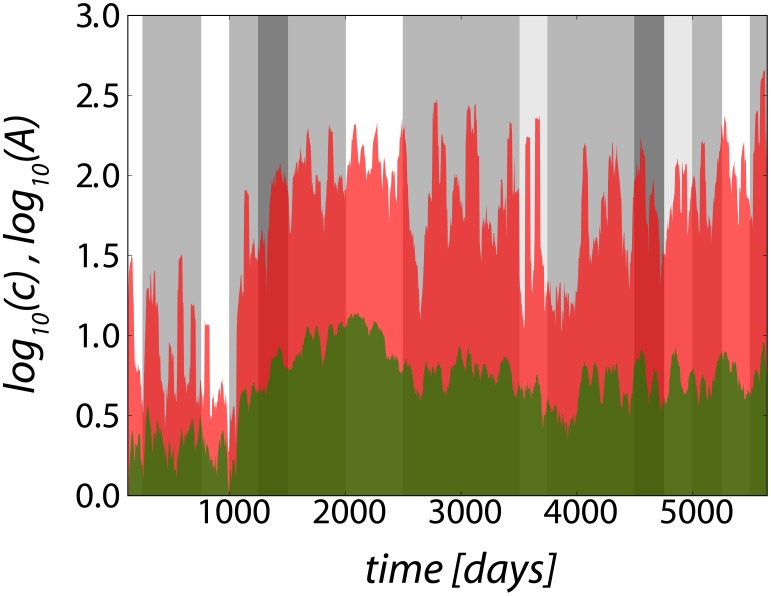
Typical time series of open source software development (e.g. Apache Web Server) with active contributors (green area) and their productive activity (red area). For clarity, the time series are represented in logarithmic scale and they have been smoothed with a rolling window of 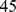 days. Over the whole project history, various epochs of productive activity can be found. The background grey areas indicate three levels of the productivity exponent 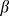 defined by equation (1) (light grey for 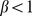, grey for 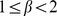 and dark grey for 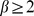) for time windows of 250 days. Blank areas show time windows for which 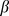 could not be fitted, mainly because the numbers of active contributors (resp. commits) were strongly varying over these periods. In other words, it is possible that super linear production was occurring in these periods but we could not determined it.

A first idea would be to quantify the total production (for instance proxied by the number of lines of code, commits or the number of packages) of each software and search for a relationship with the total number of involved developers over the whole project. This is misleading because the total output results from a complex interplay between a time varying numbers of involved developers and the intermittent duration and intensity of their contributions. In the extreme limit, a single developer working over a lifetime may produce as much as tens or even hundreds of developers over a few months. The large variability of developer numbers and contributions as a function of time for each project is illustrated by Figure 2, which shows the intermittent dynamics of active contributors as well as their productive activity as a function of time (in logarithmic scales).

To capture more faithfully the actions of contributions via cooperation, we propose to focus on short-term production and group sizes. For each project, we partition its lifetime in time windows of a fixed size that we shift over the whole project duration. We then quantify the production in each window and study its relation to the number of active developers during that same time window. As proxies for the production of developers, we could use either use lines of codes (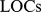) or commits. 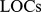 are straightforward metrics but suffer from the criticism that real production and quality is not in general proportional to the number of code lines. Indeed, excellent contributions are in general characterized by efficient and elegant coding associated with conciseness. Among software developers, it is well recognized that the number of LOCs contributed is not a predictor of quality. However, in open collaboration, each innovation step can be seen as a commit uploaded and compounded on an online repository, which keeps track of all changes over time. Each commit reflects the contributor's *commitment* to expose to the community her proposed solution to an open problem. Commits are the elementary units that get peer-reviewed, tested and eventually integrated in the project knowledge base. Thus, they are a direct measure of the iterative productive process at work in peer-production. All commit activities are parsimoniously indexed and timestamped on the project repository.

**Figure 3:**
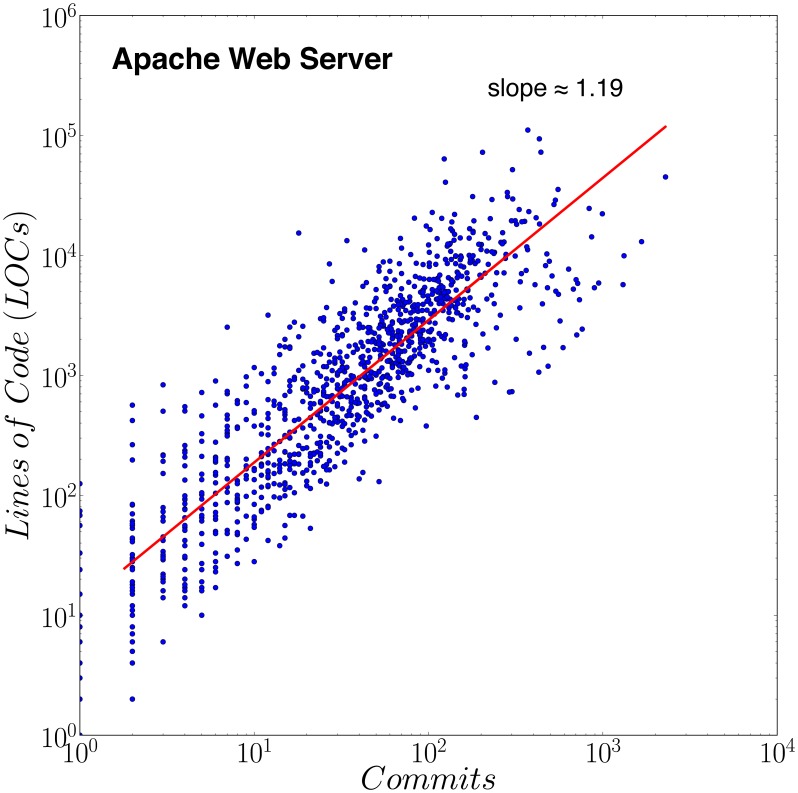
Scaling relation 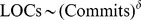 between commits and lines of code. For the Apache Web Server project, the scaling exponent is 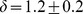 (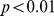, 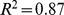). For the vast majority of projects, the relation between lines of code and commits exhibits the same scaling with 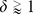, suggesting that we can use either commits or lines of codes, as both provide a consistent and therefore robust measure of contribution (and in addition that commits may themselves result from cascades of code production.

Notwithstanding these arguments in favor of using commits as metrics of production, it is useful to test for a possible relation between 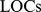 and 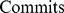. Figure 3 documents a robust scaling relationship 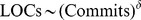, with exponents 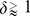 for most of the projects. These findings shown in Figure 3 bolster our confidence in the robustness of the findings reported below, which should not be sensitive to the specific choice of the metric for production.

**Figure 4:**
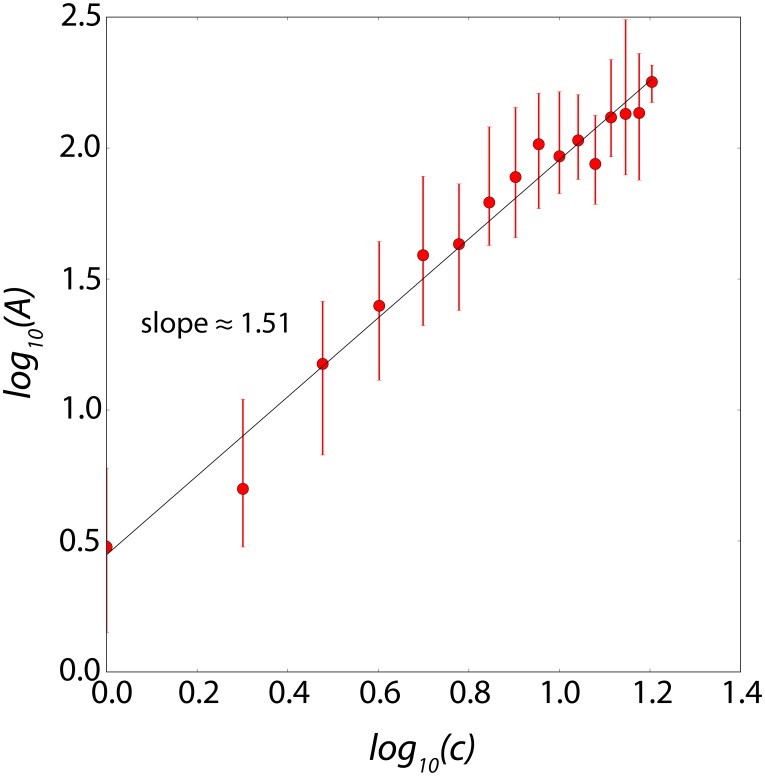
Typical superlinear relation in double logarithmic scale of the productive contribution 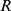 as a function of active contributors 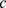 per 5-day time windows for Apache Web Server (http://httpd.apache.org/). The scaling exponent 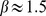 (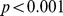 and 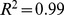) is shown as the slope of a straight line in double logarithmic scale. The error bars show the 25th and 75th percentiles of contributors log-bins.



Figure 4 demonstrates the typical superlinear relationship

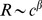
(1)where the production 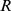 is defined as the total number of commits measured per 5-day time windows for the Apache Web Server (http://httpd.apache.org/) and 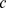 is the number of active contributors in the same 5-day time windows. Contrary to the naive expectation that the production 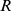 should be proportional to the number 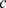 of developers, Figure 4 documents a superlinear relationship with exponent 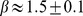, therefore significantly larger than the value 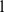 describing a simple proportionality 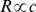. Over all OSS projects studied, the estimated statistical average is 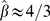. Since 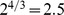, this explains the title of this paper. For many projects, 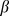 is larger than 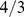, such as the Apache Web Server project shown in figure 4, for which 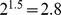. These results are robust with respect to the length of the time windows (from 1 day to 10 days).

## Mechanisms for superlinear production

We consider two classes of mechanisms for superlinear production.

### Interaction-based mechanism for superlinear production

There is a variety of channels by which contributors commit more solutions to problems when the community is more active. The peer-review process is more likely to occur when more contributors are active, there are incentives to share early with the community to avoid redundant work and some problems require collective intelligence to increase their chance to be solved [20], because they require tight coordination among different technical parts of the code [21]. A priori, the number of active developers is an extensive variable, that is, it is additive for independent non-interacting systems. When interactions between developers occur, the observed increasing return of productive activity implies that the change 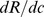 of productivity upon the addition of a developer due to the existence of interactions is not a constant but grows itself with the number of active contributors (as 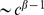 with 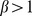). There is thus a remarkable increase of *productive activity*, not only as the sum of increased individual commits, but also as a result of interactions among active contributors.

### Interactions leading to a phase transition

In standard models of interaction, linearity between the observable and external driving field as well as number of elements in the system is the rule (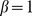), except at or close to a critical phase transition point. As an illustration, consider the average magnetisation 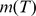 per spin at a function of the temperature 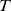 in a system undergoing a paramagnetic-ferromagnetic phase transition at the critical temperature 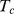. The standard relation 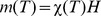 relates linearly the average magnetisation 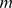 to the external intensive magnetic field 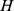 via the susceptibility 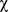. Introducing the spatial spin-spin correlation length 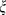 of the system, it is known that the susceptibility diverges as a power of the correlation length as 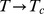


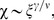
(2)where 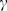 and 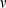 are two critical exponents related by the hyperscaling relation 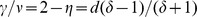, where 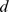 is the space dimension. Exactly at 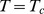, the linear relationship between 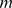 and 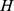 given by (2) is replaced by the nonlinear relation 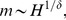
(3)defining the exponent 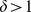. This means that the collective behaviour of the spin at criticality induces a nonlinear response of the magnetisation 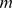 for very small external magnetic fields 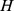 (indeed, 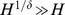 for 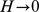 and 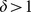). The values of the exponents are 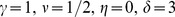 in the mean-field regime, which holds at the upper critical dimension 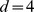. The relationship (3) looks superficially similar to (1) when compared with the standard linear relation 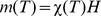, but here the magnetic field is an intensive quantity while relation (1) describes the production intensity as a function of the number of group members, which is an extensive quantity. Actually, a relation similar to (1) can be derived by introducing the finiteness of the spin system and using the theory of finite-size scaling [22]. For a system of finite linear size 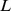 and thus finite volume 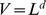, the theory of finite-size critical phenomena implied that relation (2) is replaced by 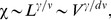
(4)obtained simply by replacing 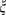 by 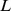. In words, the unique relevant length, which is the correlation length 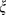 for an infinite system at criticality, becomes the system size. With 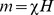, this yields 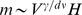. Since 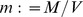 is the magnetisation per spin, we obtain that the total magnetisation 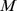 of the system with a total number 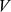 of spins is given by 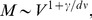
(5)that it, becomes superlinear at or close to criticality, similarly to expression (1). This type of superlinear relationship (5) holds more generally in various models of interacting elements at or close to criticality [23]–[26]. The meaning of *criticality* is that, on average, one action triggers on average one follow-up action, ensuring that the dynamics remains delicately poised between growth and decay, or between order and disorder. Therefore, an explanation of superlinear productivity by the interaction-based mechanism requires elucidating under which circumstances open source projects operate close to or at criticality. The study of dynamics of book sales [27], [28] and YouTube videos views [29] has shown evidence of these critical triggering effects in large social networks. Open source projects and their online communication platforms coupled with the code repository serve a similar social network role yet at much smaller scales [30], [31]. Since these above analyses as well as those presented here benefit from the survival bias, in other words the analyses are performed on top performers among a much larger database, the existence of criticality in these system can be interpreted as the signature of a degree of success quantified by significant activity. Specifically, considering a large universe of projects, those that are of interest in the sense of exhibiting significant dynamics in volume and quality are those for which the conditions are met to be close to criticality.

### Interactions leading to superradiance-like phenomena

The superlinear dependence of the production intensity as a function of the number of group members has a rather direct analog with the phenomenon of superradiance [32], [33], a coherent effect in many-body systems of 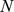 excited emitters that interact with a common light field. In the limit when the wavelength of the light is much greater than the separation of the emitters, then the emitters interact with the light in a collective and coherent fashion. Rather than radiating independently with a total intensity proportional to 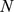 as would be expected for independent emitters, in the most favorable case of perfect coherence, the total radiation scales as 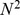, similarly to the mean-field prediction 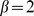 obtained from expression (13) when the exponent 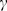 of the tail distribution of first generation contributions per developers is larger than or equal to 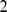. For more realistic experimental situations, the exponent is smaller than 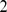, for instance equal to 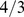 when the initial light fluctuating field is small [34], or equal to 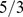 for 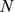 two-level atoms placed within isotropic photonic band-gap material (but can reach the value 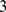 for anisotropic 3D band gaps) [35]. In physics, the superradiance effect results from the existence of correlations and interactions between emitters, similarly to the interactions between group members of OSS projects. The interactions and resulting correlations between emitters are mediated by the radiated light, similarly to the correlations between developers via the production of commits. The superradiant emission is a cooperative process involving a collective mode of all the atoms of the sample. In this collective mode, an “order” appears in the system which can be defined by the build up of correlation between the dipoles belonging to different atoms. This correlation is quite reminiscent of the spin-spin correlation appearing for example in a ferromagnetic sample [33]. There is in fact a hidden phase transition in which the role of the diverging correlation length is played by the light wavelength, which has to be much larger than the inter-emitter distances.

Moreover, the smaller value of the exponent 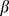 for large groups and for cities, as documented below, has a straightforward interpretation in the superradiance analogy. Indeed, the maximal number of correlated emitters is limited by the correlation, or coherence volume. When the number of emitters exceeds the maximal number of those that effectively interact, the superlinear exponent decreases. This is due to the fact that, for larger numbers of emitters, the system separates into clusters or subgroups that radiate practically independently. In physics, this effect is termed filamentation. The same effect is argued to happen for the studied case of production intensity, as is discussed in the section below entitled “Reconciling present findings and superlinear production in large cities”.

## Large deviation mechanism for superlinear production

**Figure 5:**
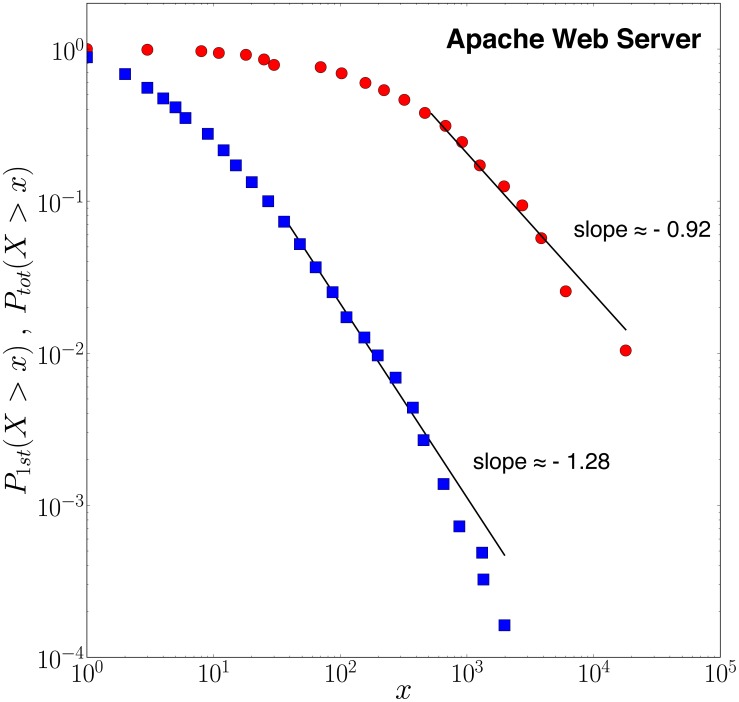
Typical distributions of 1^st^ generation daughter events and total number of commits per developer for the Apache Web Server project: (blue squares) Complementary cumulative distribution 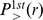 of contributions (number of commits) per developer and per 5-day time bins (1^st^ generation daughters events in the language of the epidemic branching process described in the text) with exponent 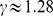. (**red circles**) Complementary cumulative distribution 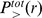 of all contributions per developer over a long period of time. 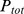 is equivalent to measuring the cluster sizes of contributions following critical cascades (7). All distributions have been fitted using the maximum likelihood estimator (MLE). The distribution of cascade size is characterized by the exponent 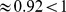 compared to the first generation daughter events distribution with exponent 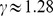. The results showed here for Apache are representative of the distributions found in other collaborative projects.



The second class of mechanisms builds on the evidence of large deviations in the statistics of the production activity 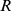 over the whole population of contributors and over the whole life of the project. Figure 5 shows the complementary cumulative distribution 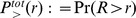 of all contributions per developer over a long period for the Apache Web Server project. One can observe an approximate power law tail dependence

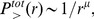
(6)with 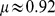. Within the epidemic framework presented in the next section, 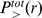 will be shown to be equivalent to the statistics of the cluster sizes of contributions following critical cascades [36] (see expression (12)), i.e., when the dynamics of triggering of activity is close to or at the critical point of a branching process. This result, showed for the Apache Web Server project, is representative of the distributions found in other collaborative projects.

In the presence of such a power law statistics of contributions characterized by an exponent 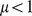, we show below that the sum of contributions over all developers is controlled by extreme contributors. The contributions made by these exceptional members of the group are also responsible for the observed superlinear behavior given by (1). This mechanism is reminiscent of the improved group performance that results from the presence of one or few surperforming individuals [37]. In this case, the largest contributor provides a finite fraction of the whole production over a given time period. This largest contributor (i.e. the “large deviation”) has a superlinear contribution in the group size [38], [39]. In this situation, the increasing productive activity results from a large heterogeneity of activity per individual. And the more contributors 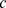 during a production period, the more likely it is to find an extremely large contribution.

Specifically, starting from expression (6) for the complementary cumulative distribution 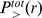, we denote 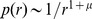 the corresponding probability density function obtained as the derivative of 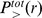. Let us call 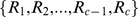, the total number of commits contributed respectively by the developers 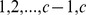. Let us call 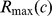, the largest among the set 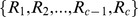. A good estimate of 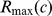 is obtained by the condition that the probability 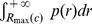 to find a developer with a total contribution equal to or larger than 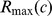 times the number 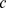 of active developers is equal to 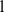, i.e., by the definition of 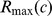, there should be typically only one developer with such a number of commits. This yields 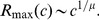
(7)


An estimate of the typical total number of commits 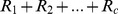 contributed by the 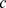 developers can then be obtained as [38], [39]

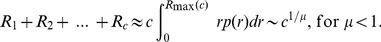
(8)


We stress that the scaling 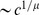 only holds for 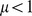 and is replaced by 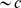, i.e., linearity, for 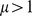. The upper bound in the integral in (8) reflects that the random variables 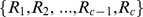 are not larger than 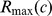 by definition of the later. According to equation (8), the typical total production (number of commits) by 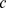 developers is proportional to 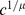, when their contributions are wildly distributed with a power law distribution with exponent 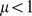. According to this large deviation mechanism, the superlinear exponent 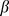 is equal to 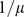. 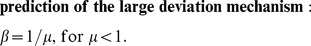
(9)


Within this large deviation mechanism, explaining the superlinear productive activity (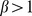) reduces to explaining the heavy-tailed distribution of commits 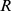 per contributor over a large period of time, i.e., amounts to derive the power law distribution (6) with 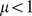. For this, the next section proposes a generic model.

## Cascading model of productive activity

Both the *interaction-based* and the *large deviations* mechanisms can be captured together by a generic cascade process, which is well described by the excited Hawkes conditional Poisson process [40]. The Hawkes process typically models well a variety of social dynamics involving complex human interactions such as online viral meme propagation [29], gangs and crime in large American cities [41], cyber crime [42] and financial contagion [43]–[45]. The Hawkes process is defined by the intensity 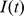 of events (commits) given by 
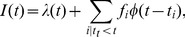
(10)where 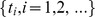 are the timestamps of past commits, 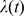 is the spontaneous exogenous rate of commits, 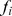 is the fertility of commit 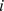 that quantifies the number of commits (of first generation) that it can potentially trigger directly, and 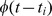 is the memory kernel, whose integral is normalized to 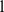, which weights how much past commit activities influence future ones. The function 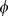 typically reflects how tasks are prioritized and performed by individuals according to a rational economy where time is a non storable resource [46]. Expression (10) expresses that the number of commits contributed between time 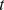 and 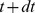 results from two sources: (i) an exogenous source 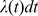 representing the spontaneous commits not related to previous commits; (ii) an endogenous term represented by the sum over all commits that were made prior to 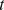, and which are susceptible to trigger future commits. An obvious triggering mechanism is debugging: a past commit may attract the attention of a developer who fixes a bug and thus improves the code. Another triggering mechanism by which a previous commit may trigger a future commit is when the former enables new functionalities and relationships that open novel options for the developers. The Hawkes model is the simplest conditional Poisson process that combines both exogeneity and endogeneity.

The class of Hawkes models can be mapped onto the general class of branching processes [47]. The statistical average fertility 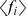 defines the branching ratio 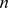, which is the key parameter. For 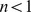, 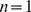 and 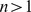, the process is respectively sub-critical, critical and super-critical [48], [49]. In the sub-critical regime (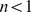), the average activity tends to die out exponentially fast and the exogenous source term 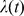 controls the overall dynamics. At criticality (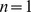), on average one commit is triggered in direct lineage by a previous commit, corresponding to a marginal sustainability of the process with infinitesimal exogenous inputs. The super-critical regime (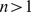) is characterised by an explosive activity that can occur with finite probability. The results derived below are thus fundamentally associated with the existence of a critical phase transition determined by the control variable 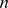. The nature of the critical phase transition for this Hawkes model with distribution of fertilities has been described in Refs. [36], [50], [51]. Interpreting a cluster or connected cascade in a given branching process of triggered contributions as the burst of production in a group of developers, the distribution of contributions is thus mapped onto that of triggered cluster sizes [36].

Let us define the complementary cumulative distribution 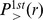 of contributions (number of commits) per developer directly triggered by a given past commit, which can be called first-generation *daughter* commits generated by a *mother* commit. Consider the case where 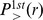 is also a power law 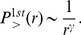
(11)


Close to or at criticality, the distribution of cluster sizes, which is equivalent to the distribution of productive activity 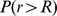 given by (6) has an exponent 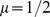 [52], under the condition that the distribution 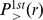 of contribution sizes triggered directly by previous contributions (so-called first-generation cascades) decays sufficiently fast, i.e., with 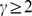. The result 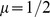 holds also for any distribution 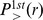 decaying asymptotically faster than a power law [36]. When 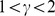, the mean field exponent 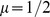 is changed into [36]

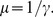
(12)


Together with (9), the superlinear exponent 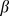 is predicted to be 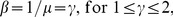
(13)that is, equal the exponent 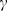 of the tail distribution of first generation contributions per developers. For 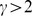, 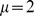 and therefore 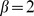. An analytical derivation of the prediction (13) using the Hawkes process (10) that anchors rigorously the large deviation argument of the previous section is given by Saichev and Sornette [53].

**Figure 6:**
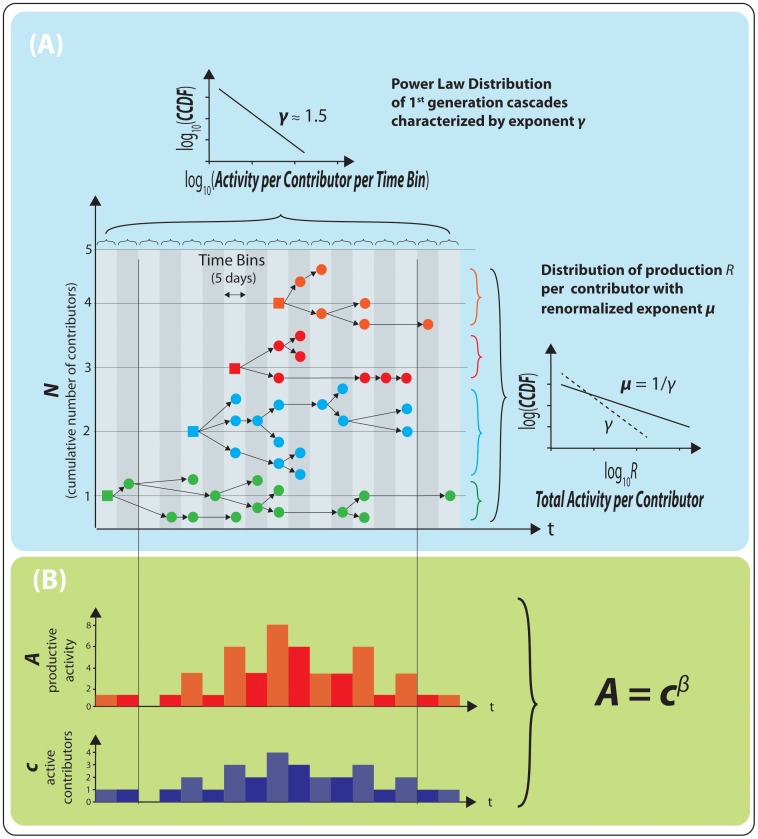
Relationship between superlinear productive bursts, cascading dynamics, and heavy-tailed distributions of 1^st^ generation and cumulative contributions. (**A**) (light blue) Triggering mechanism generating the clusters of size with renormalized exponent 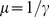 from the distribution of first generation “daughter events” with exponent 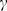. For the sake of simplicity, we represented one cluster of activity per contributor, but triggering can occur between contributors provided that the probability of triggering remains the same between all contributors. (**B**) (light green) shows how the triggering mechanism generates superlinear productive activity 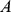 as a function of the number of active contributors 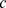.

Figure 6 synthesizes the relation between superlinear productive activity, (critical) cascades, the distribution of first-generation triggering and the total distribution of activity per contributors over a sufficient long period.

## Empirical tests

We now turn to empirical tests of this theory. For each 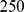 days period and for each project in our database (Archive S1), we have calibrated the power law tails of two distributions:

1. the distribution of the total number 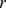 of commits per contributor over the 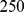 days, which is taken as a proxy for 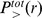, with exponent 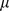;
2. the distribution of the number of commits per developer per 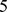 days time bin, which is assumed to be a reasonable proxy for the distribution 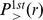 of the first generation production characterized by the exponent 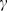.

For each OSS project, we have used the discrete maximum likelihood estimator (MLE) with a p-value threshold 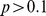, obtained by bootstrapping, and Kolmogorov-Smirnov Distance 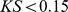 to select the ranges over which the calibration is performed [54] (see Table S1, for detailed results of each OSS project analyzed).

**Figure 7:**
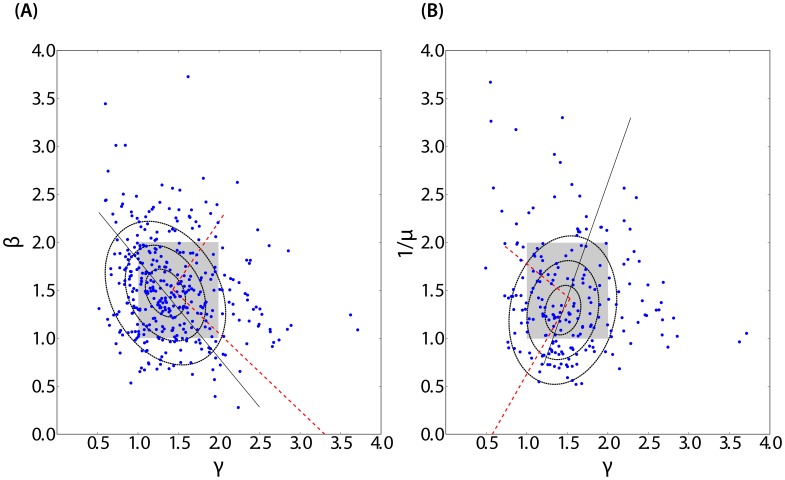
Verification of the relationship between 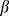, 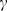 and 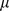 as predicted by the theory. (**A**) superlinear exponent 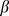 as a function of 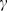, the exponent of the power law tail distribution of first generation productivity for each of the 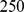 days periods for which both values could be calibrated. The points are concentrated around 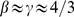 with almost half of them (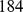 over 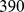 values) within the grey area delimited by 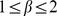 and 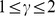. To test for the relations 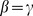 and 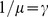, we used a bi-Gaussian model. The dotted ellipses show the first three standard deviations around the barycenters and the black line represents the main axis with the bi-Gaussian model. We also performed a principal component analysis (PCA). The red dotted lines show the main direction of variance obtained with the PCA. Both methods show a positive relation between 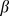 and 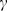 only on second principal component (slope 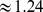 with PCA). (**B**) same as panel (A) for the dependence of 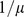 versus 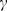 with a concentration of points in the grey area (86 over 213 values) and 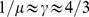. Both the bi-Gaussian fit and the PCA show strong evidence of a positive relation with slope 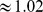 with the bi-Gaussian approach and 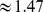 with the PCA.

Figure 5 shows the result for the Apache Web Server project. The fitting procedure qualifies the existence of a power law tail for the two empirical distributions with estimated exponents respectively equal to 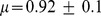 and 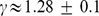. These values with their error bars are compatible with the prediction (12) 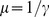, resulting from the cascades of triggering [36]. This result is typical of the other investigated OSS projects, as shown Figure 7, albeit with a considerable variability. This is expected since the projects are likely to be characterized by many more dimensions that the production and cascading effects considered here.

Figure 7 presents 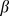 as a function of 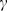 (panel A) and 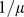 as a function of 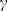 (panel B) for all the OSS projects on our database, According to the cascading model of productive activity presented in the previous section, we should have 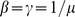, according to (13). Indeed, one can see that 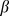, 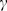, and 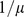 are clustered around 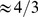. Almost half of the considered periods (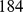 of a total of 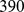) fitted over all projects belong to the regime where 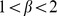 and 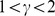 (panel A) and forty percent (86 out of 213) are such that 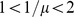 (panel B) as predicted by the theory.

Let us first focus on the relationship between 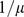 and 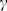 shown in panel B of Figure 7. Note that the statistics on the exponent 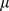 is significantly smaller compared to that for 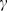 simply because we obtain one data point over each 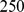 day periods for 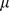 compared with one data point per 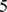 days time bin for 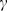. The shaded square represents the domain over which the theory applies (86 over 213 data points). To test quantitatively the relation 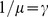, we used a Gaussian bivariate distribution model. The dotted ellipses show the first three standard deviations equi-levels around the barycenter 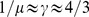 and the black line represents the principal axis of the bi-Gaussian model. We also performed a principal component analysis (PCA). The red dotted lines show the two main directions of the variance obtained with the PCA. Both methods support a positive correlation between 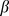 and 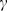 with slope 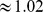 with the bi-Gaussian approach and 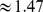 with PCA. To our knowledge, this may be the first empirical test ever of the renormalization of the exponent 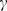 of first generation events into the renormalized exponent 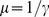 due to the cascade of triggering over all generation in a critical branching process [36], [52].

The evidence for the relationship between 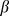 and 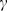 is presented in panel A of Figure 7. First, one can observe a prevalence of the large-deviation critical interaction regime as the grey square area delimited by 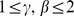 is very densely populated (184 out of 390). Second, as already pointed out, the barycenter of the cloud of data points is on 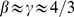, as expected from theory. However, we find limited support for a clear linear relation between 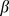 and 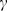. The bi-Gaussian model analysis provides the three dotted ellipses showing the first three standard deviations away from the barycenter. The black line representing the main axis of the bi-Gaussian model suggests a negative correlation between 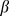 and 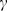. Using a PCA analysis, we find a positive relationship on the second principal component, with slope 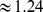. These results suggest that very productive projects and periods within projects, characterized by a large superlinear exponent 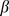, are likely to be due to more complex interactions between the developers and their mutual triggering that assumed by the simple theory developed above. In particular, differentiation between same-developer commit triggering and inter-developer commit triggering seem necessary along the lines of Refs. [19], [55].

## Reconciling present findings and superlinear production in large cities

**Figure 8:**
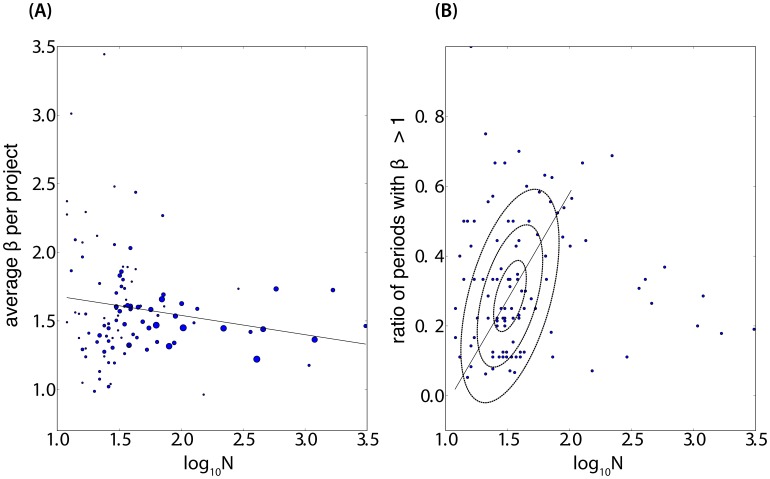
Evolution of the superlinear exponent 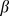 as a function of project size. (**A**) Average superlinear exponent 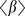 per project as a function of the cumulative number of contributors. The circle size reflects the number of exponents fitted per 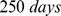 time window, for each project and entering the average statistics. The sampling ranges from 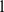 (small disks) to 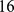 (largest disk). 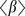 exhibits a slightly negative slope 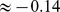 as a function of 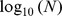 (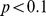 and 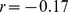). (**B**) To measure the prevalence of productive bursts in projects, we measure the ratio of periods with superlinear exponent 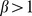 over all 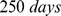 periods for each project as a function of 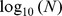. We distinguish a cluster of points around 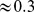 and 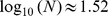 (i.e. 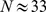 contributors) with a positive relationship (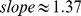) of the ratio as a function of 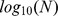. Projects with a large pool of contributors (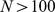) are more randomly scattered with a lower ratio and do not obey the same relationship, suggesting a different regime.

Figure 8 reveals that the clouds of superlinear production exponent 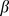 exhibit an interesting regularity as a function of the total number of contributors 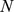 of an OSS project. The intuition motivating this investigation is the following. While a minimum critical mass of contributors is needed to foster productive bursts, large projects suffer from coordination costs, which may offset the increasing return of productive activity. Figure 8 (panel A) shows indeed that the superlinear exponent 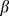 decreases on average with the size of the projects. Panel B demonstrates that, for projects of up to 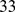 contributors, the number of 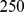 days periods with 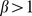 (superlinear regime) increases as a function of the total number 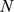 of developers, approximately according to 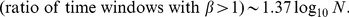
(14)


For 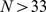, a different regime occurs characterized by a much smaller ratio of the time periods with superlinear productivity (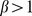). Taken together, the two panels of Figure 8 support the view that superlinear productivity is the appanage of relatively small projects with no more than 30–40 developers in total, while larger groups face the difficult challenge of creating and maintaining productive bursts. The data is too scattered unfortunately to allow us to draw a firm conclusion on the value(s) that 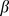 converges towards for large project sizes.

There may be a link between our results and a previous study reporting the phenomenon of superlinearity on a completely different class of objects, namely cities. Data from 360 US metropolitan areas have shown that wages, number of patents, GDP and intensity of crime scale superlinearly with population size [production 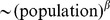] with an exponent 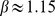 [56], [57]. The value of 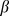 larger than 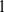 reflects the fact that productivity increases by about 11% with each doubling in population [58]. Qualitatively in line with our findings, the superlinearity found in our OSS data is significantly stronger (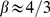 on average, with large variations and some projects being characterised by much larger 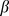
's) for the smaller projects with no more than 30–40 developers. We note that our results apply to a completely different range of group sizes compared with the results for cities involving population of tens of thousand to tens of millions inhabitants.

The underlying mechanisms are perhaps different [59]. For cities, the superlinear scaling in urban productivity demonstrates the importance of cities as centers of enhanced interactions, leading to generation and exchange of knowledge and exploitation of innovations [58]. For the OSS projects, many other factors come into play, such as the role of diversity and complementarity, which describes the fact that doing more of one thing increases the return to doing more of another. Other possible mechanisms include synergies, economies of scale, coordination and leadership, role model and entrainment effect, motivations, friendship and other psychological factors. However, Figure 8 suggests that these mechanisms dampen out as the project size becomes very large, possibly leaving only those still active at the level of city sizes.

Expanding on the remark on the different sizes involved in our OSS database compared with cities, we present a simple mechanism and theoretical argument that may explain the smaller value of the superlinear exponent for cities, deriving it from our results obtained for small group sizes. The key idea is that the population of a city can be partitioned into many groups of persons interacting closely within a group and loosely or not at all across groups. Groups can be firms, or department within firms, clubs, and other organisations through which people interact. We assume that, within each group, the superlinear production law (1) holds with the exponent 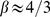 found in our OSS database.

The second ingredient is that group sizes 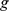 are widely distributed, roughly as Zipf's law [15], 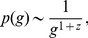
(15)where 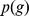 is the probability density function of the group sizes 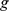, 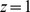 if Zipf's law holds exactly, while in general 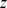 can deviate from 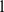 for a variety of reasons [16]. Let us assume that a city of total population 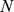 is constituted of 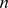 groups, respectively with memberships of 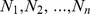 individuals. The total production of the city is then, according to (1), 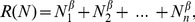
(16)assuming for the moment and for simplicity that 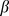 is independent of group sizes. 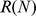 in expression (16) can be estimated as [38], [39]

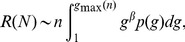
(17)where 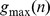 is the largest group size among the 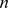 groups, which can be estimated by 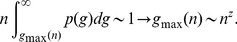
(18)


By conservation and assuming for simplicity no strong overlap between the groups, we have approximately 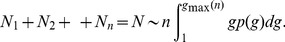
(19)


This leads to 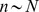 for 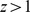 and 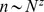 for 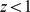. In words, a relatively thin tail of the group size distribution (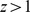) is associated with a number of group scaling proportionally to the total city population 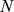. In contrast, for a heavy tailed distribution (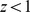), the number of groups scales sublinearly with 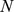, as the few largest groups account for a finite fraction of total population. Reporting in expression (17), this yields 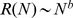, with the exponent 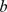 obeying three possible regimes.

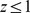
 implies 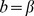: the same superlinear production exponent defines the whole city production as a function of its population as does the production of each independent group. The mechanism is clear: for 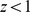, a few single largest groups dominate the 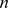-partition and account for the majority of the city population. The same scaling holds essentially because the city is almost controlled by a single group and we have assumed the same exponent 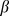 for all groups. The empirical evidence suggests that this case does not apply.

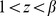
 implies 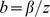. In this regime, there are still very large groups that contribute to the superlinearity but their relative numbers is much less than for 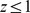. The values 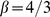 with 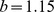 can be reconciled with 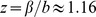. This exponent is, with error bounds, roughly compatible with the value found for firms in the US, close to 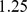 [60].

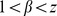
 implies 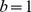, which corresponds to a linear growth of production of the city with its population. In this regime, the overall city production is controlled by the many small groups constituting the city and there are no scale effects other than a proportionality with the number of small groups.

While this argument is quite naive, it demonstrates the importance of the interplay between partitions of cities in groups, the corresponding productivity of such groups and the size distribution of these groups. A similar story is likely to be relevant in large OSS projects, groups and firms, which for a variety of reasons ranging from cognitive limitations [61] to efficiency maximization [62] are found to organize in subgroups, often in a hierarchical way [61].

## Discussion

In the early days of the industrial revolution, Adam Smith noted how the successive efficiency gains of communication means have helped reach unprecedented pools of resources and how they have unlocked some limitations of the labor market through improved division of labor [63]. The telegraph, telephone and more recently the Internet have further pushed back the possibilities for knowledge production and for labor organizations on the model of collective action [64]. Nowadays, unrelated people spontaneously team up across the world in open collaboration projects and join forces to create knowledge in the form of software, natural language [65], mathematics [66] as well as for the production of tangible goods [67]. These organizations rely primarily on the principles of peer-production [68]: (i) task self-selection, (ii) peer-review and (iii) iterative improvement, at odds with traditional market and firm production organizations [69]. Expertise can be timely and rightly pulled from a broader community towards efficient problem resolution. The present understanding of group performance in social psychology goes in the same direction: experiments involving small groups performing coordination tasks [8], [70], problem solving [37] and innovation [14] support the hypothesis that larger groups perform better because more diverse cognitive abilities can be pooled. Group productive activity can also be *more than the sum of their parts* if members develop social sensitivity among each others [20]. However, the marginal gain of having more individuals in a group decreases rapidly to be negligible beyond five individuals [37], [71], [72]. Similarly, as projects attract larger communities, more coordination is required through social norms and formal governance structures [21], which may in turn reduce the positive effects of peer-production [73].

## Conclusion

In this paper, we have shown that productive bursts, associated with increasing return of activity, result from the mechanism of critical triggering of commits among contributors. Specifically, we have shown that production intensity, or production per unit time, grows superlinearly as a function of the number of participants in a group. Practically, we have found a superlinear relationship 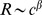 with 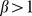 between the total number 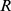 of commits measured per 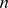-day time windows for different OSS projects and 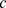 is the number of active contributors in the same 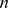-day time windows. We have found that these results are robust with respect to the length 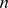 of the time windows, i.e. when varying 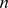 from 1 day to 10 days.

Such critical triggering may operate according two co-existing mechanisms: *interactions* and *large deviations*. These mechanisms have been falsified in three independent ways: (i) documenting the superlinear relationship between productive activity 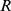 and the number of active contributors 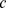 characterized by the scaling exponent 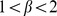; (ii) measuring the power law tail distribution of first generation cascades with exponent 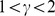 and checking that it explains the superlinear productivity exponent 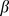; and (iii) measuring the power law tail distribution of production cluster sizes with exponent 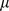 and verifying that it is approximately equal to the 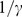, where 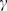 is the distribution of contributions per developer at short times.

We have found that superlinear productive activity holds for a broad range of project sizes and types, with a slight decrease of the average scaling exponent 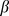 with the total number of contributors 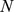. The frequency of productive bursts occurrence in projects has been found to be very large for 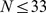 compared with larger projects. The results suggest that size and threshold effects have an influence on the ability to trigger and maintain critical triggering of individual contributions. Indeed, contributions must create enough reaction opportunities to trigger on average as many follow-up contributions. Pervasive communication systems (social networks), physical proximity (e.g. cities), or even personal dedication to the project surely help increase opportunities for a contribution to trigger a follow-up action. On the other hand, large and complex structures with overwhelming communication loads or inadequate governance structure can inhibit the *ripe* circulation and reuse of knowledge for the sake of further cumulative innovation. The *large deviation* mechanism provides another take-away lesson: open collaboration does not imply equal work between contributors. On the contrary, productive bursts are the hallmark of a minority of individual engagement with intense interactions and short-lived contributions of far above average sizes. Whether these large deviation contributions pull engagement by others or on the contrary are pushed by the community remains an open question to be elucidated.

## Supporting Information

Table S1Table containing summary statistics (comma separated file), 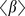, 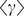, and 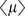, for each project analyzed in this study.(CSV)

Archive S1 **Compressed archive of Python Numpy arrays containing the time series of all commits, including timestamp, user, file modified, for each open source software project analyzed in this study.**
(ZIP)
